# Treatment Approach for Advanced Systemic Light Chain Amyloidosis: A Case Report

**DOI:** 10.7759/cureus.65960

**Published:** 2024-08-01

**Authors:** Mohamad Ali M Hachem, Ghadir M Nasreddine, Solay Farhat, Zeinab M Hammoud, Firas Saad, Wajih A Saad

**Affiliations:** 1 Hematology and Oncology, Faculty of Medical Sciences, Lebanese University, Beirut, LBN; 2 Hematology and Oncology, Morristown Medical Center, Morristown, USA; 3 Oncology, Faculty of Medicine, Lebanese University, Beirut, LBN

**Keywords:** case report, bortezomib, cyclophosphamide, daratumumab, light chain amyloidosis

## Abstract

Systemic light chain amyloidosis is a rare and severe disorder characterized by amyloid fibril deposition in various tissues, often leading to organ failure. Early diagnosis is crucial but challenging due to diverse clinical manifestations. Our case report presents a complex case of systemic light chain amyloidosis in a 62-year-old patient with cardiac, renal, neurological, and gastrointestinal involvement. The patient's treatment with cyclophosphamide, bortezomib, dexamethasone, and intravenous daratumumab yielded significant improvement, aligning with recent studies. Following treatment, the patient improved from stage IV to stage II systemic light chain amyloidosis per the National Comprehensive Cancer Network (NCCN) guidelines, indicating a more favorable prognosis. Hence, the successful integration of daratumumab in our case underscores its potential as a valuable addition to the treatment regimen for advanced systemic light chain amyloidosis, showcasing significant improvements across multiple organ systems.

## Introduction

Amyloidosis is a group of disorders characterized by the aberrant malformation of proteins, resulting in the deposition of insoluble fibrils in diverse tissues of the human body [1]. Among the different types of amyloidosis, systemic immunoglobulin light chain amyloidosis (AL amyloidosis) prevails as the most frequent form, accounting for about 70% of all amyloidosis cases [2]. AL amyloidosis presents as a multifaceted condition driven by the anomalous proliferation of malignant plasma cells, generating atypical immunoglobulin light chains that aggregate to form amyloid fibrils [3]. These amyloid fibrils infiltrate and damage various organs, most commonly the heart, kidneys, and nervous system, leading to progressive organ dysfunction and eventually death [4]. Indeed, this condition represents a significant challenge in clinical practice due to its diverse clinical manifestations and potential for rapid progression. Despite its rarity, with a worldwide incidence ranging from 5.1 to 12.8 cases per million person-years, the disease carries substantial morbidity and mortality as well, leading up to 24-37% of patient deaths within six months of diagnosis [5]. In the United States alone, between 1,275 and 3,200 new cases are diagnosed annually, underscoring the significance of this condition [4]. It is important to note that the clinical presentation of AL amyloidosis is highly heterogeneous and largely depends on the organs affected by amyloid deposition. Hence, patients may present with a spectrum of symptoms, ranging from nonspecific fatigue and weight loss to organ-specific manifestations such as congestive heart failure, proteinuria, neuropathy, and hepatomegaly [6]. The diverse array of clinical manifestations often complicates the timely diagnosis and intervention of this condition, often contributing to its rapid progression. In fact, the management of AL amyloidosis remains challenging and requires a tailored approach based on disease severity, organ involvement, and patient-related factors. This case report aims to elucidate a comprehensive treatment approach for AL amyloidosis, offering valuable insights into the management of this challenging condition.

## Case presentation

This case report presents a case of a 62-year-old male patient with no previous medical history who presented with the chief complaint of syncope. During history taking, the patient reported dyspnea, orthostatic hypotension, fatigue, vertigo, gastroparesis, urinary incontinence, and alternating recurrent episodes of constipation and severe diarrhea. On physical examination, the patient presented with lower limb edema pitting 2+, peripheral neuropathy, and decreased bilateral airway entry at the lung base on auscultation. Several tests were conducted during the diagnosis process. Upon presentation, the patient had borderline systolic blood pressure (SBP) 100 mmHg. 

Initially, blood workup showed normocytic anemia in the complete blood count (CBC) (Hgb = 10 g/dL, mean corpuscular volume (MCV) = 85 fL). Basic metabolic tests reported severe hypokalemia (K = 2.7 mmol/L), normal creatinine (0.8 mg/dL), and severe hypoalbuminemia (Alb = 1.5 g/dL). Inflammatory tests showed an elevated erythrocyte sedimentation rate (ESR) of 35 mm/h. A CT scan of the chest, abdomen, and pelvis revealed bilateral pleural effusion, mild pericardial effusion, no hepatosplenomegaly, mild ascites, gastric wall thickening, and multiple rib fractures, attributed to recurrent falls due to multiple syncope episodes. Furthermore, kidney assessment was done, and urine analysis revealed the presence of proteinuria (++++), with a volume of 750 mL/24 hours and 4,020 mg of protein in a 24-hour urine collection. Alongside these significant findings, free kappa light chain was 18.56 mg/L and free lambda light chain was elevated at 215.36 mg/L, with an abnormal kappa/lambda ratio = 0.09 (<0.26) and differential free light chain (dFLC) = 197 mg/L (>180 mg/L). Also, urine protein electrophoresis (UPEP) with immunofixation showed Bence Jones proteinuria of the free lambda type, and serum protein electrophoresis (SPEP) with immunofixation revealed the presence of a monoclonal peak of the free lambda type in the beta region (1.4 g/L). Therefore, a bone marrow aspirate was done, with the presence of phenotypically suspicious clonal plasma cells (12%) on medullogram and flow cytometry. Molecular analysis with fluorescence in situ hybridization (FISH) was negative for genetic abnormalities. The diagnosis of AL amyloidosis was established through tissue biopsy, which revealed amorphous eosinophilic deposits with apple-green birefringence under Congo red staining, indicating amyloid fibrils. Immunohistochemistry confirmed the overexpression of lambda light chains, consistent with AL amyloidosis. A bone marrow biopsy showed 12% clonal plasma cells, but it is crucial to note that monoclonal gammopathy and elevated serum lambda light chains alone do not confirm AL amyloidosis. It is acknowledged that other types of amyloidosis could coexist with monoclonal gammopathy of undetermined significance (MGUS). However, the combination of tissue biopsy findings, clinical presentation, and laboratory results, including elevated N-terminal pro B-type natriuretic peptide (NT-proBNP) and proteinuria, strongly supports the diagnosis of AL amyloidosis in this patient. The monoclonal spike (M spike) value was 1.4 g/L, as noted in the SPEP results. This value further supports the presence of a monoclonal protein, contributing to the diagnosis.

Due to the presence of lower limb edema, dyspnea, bilateral pleural effusion, and a positive troponin level of 0.338 ng/mL, a cardiac assessment was done. Echocardiography showed left ventricle mild concentric hypertrophy with a normal-sized cavity, mild pericardial effusion, and an ejection fraction of 55%. Also, NT-proBNP level was very elevated (7166 pg/mL), and BNP level was increased (453 pg/mL). These findings of unexplained nephrotic-range proteinuria, Bence Jones proteinuria on UPEP, monoclonal peak on SPEP, along with an increase in NT-proBNP, in the absence of known primary heart disease, and concentric left ventricular hypertrophy on echocardiography raise suspicion of systemic light chain amyloidosis. To confirm the diagnosis, a tissue biopsy should be done. Due to the presence of gastric wall thickening on imaging, a gastroscopy was performed, revealing antritis. Biopsy findings indicated the presence of amorphous eosinophilic deposits, demonstrating apple-green birefringence when stained with Congo red, indicating the presence of amyloid fibrils. Immunohistochemistry revealed strong positive staining and overexpression of the lambda light chain in cells. This pattern is consistent with amyloidosis type AL. Serum amyloid A (SAA) protein was 300 mcg/mL.

In addition, the patient complains of lower limb peripheral neuropathy. EMG showed a decrease in response in both peroneal and tibial nerves bilaterally, indicating the presence of severe bilateral lower limb neuropathy. 

Upon careful consideration of the findings and adhering to the National Comprehensive Cancer Network (NCCN) Guidelines for systemic light chain amyloidosis, version 2.2023 [7], the patient’s diagnosis was established as stage IV systemic light chain amyloidosis. This diagnosis was based on the presence of three prognostic risk variables (troponin ≥0.1, BNP ≥400, NT-ProBNP ≥1,800, and dFLC ≥180 mg/L), and evidence of multi-organ involvement: cardiac (elevated NT-proBNP in the absence of renal failure), gastrointestinal (amyloid deposit in stomach), renal (proteinuria), and nervous system (peripheral neuropathy, voiding problems, and gastric emptying disorder). In line with these guidelines, a bone marrow transplant is not considered feasible due to the significant impact of this disorder on at least four organs, notably affecting the heart, nervous system, kidneys, and gastrointestinal tract. However, we plan to reassess the possibility of transplantation after undergoing systemic therapy.

As for the treatment attempt, and despite the absence of approved therapies specifically designated for light chain (AL) amyloidosis, the standard of care of utilization of cyclophosphamide, bortezomib, and dexamethasone (CyBorD) was entailed as a recognized treatment regimen. Moreover, in accordance with the study by Palladini et al., which highlights the association of CyBorD with subcutaneous daratumumab in improving organ responses among 28 newly diagnosed AL amyloidosis patients, the treatment plan for our patient was set [8]. It included six cycles of daratumumab (16 mg/kg) combined with CyBorD (cyclophosphamide 300 mg/m^2^, bortezomib 1.4 mg/m^2^, and dexamethasone 40 mg) given on days 1, 8, 15, and 22 for each cycle. Daratumumab will be administered weekly for the first two induction cycles, then biweekly from cycles 3 to 6. Noteworthy, daratumumab was administered intravenously (IV) instead of subcutaneously. Additionally, the patient received immunoglobulins at a dose of 400 mg/kg once per month due to immunosuppression observed during treatment.

Following the completion of two cycles of the aforementioned treatment, the patient exhibited significant clinical improvement. Notably, there was a significant decrease in lower limb edema and dyspnea. The frequency of episodes of diarrhea and constipation also decreased notably. Additionally, the patient’s gastroparesis displayed improvement, and there was a significant decrease in the occurrence of syncope, orthostatic hypotension, and urine incontinence. Therefore, follow-up tests were performed. A metabolic workup revealed an increase in albumin level to 2.2 g/dL. Repeated imaging showed a decrease in bilateral pleural effusion, pericardial effusion, and ascites. Additionally, kidney function improved, indicated by a reduction in proteinuria to 2,300 mg in a 24-hour urine collection. Post-treatment, the free lambda light chain normalized to 16 mg/L, with a dFLC 4 mg/L, and the kappa/lambda ratio normalized to 1.18. No Bence Jones proteinuria was found on UPEP, and SPEP showed an absence of a monoclonal lambda-type band. Surprisingly, the cardiac biomarkers improved, with troponin decreasing to 0.106 ng/mL, NT-proBNP decreasing to 5,399 pg/mL, and BNP decreasing to 165 pg/mL. An echocardiogram revealed a left ventricle of normal size and an ejection fraction of 64%. However, bilateral sensory and motor peripheral neuropathy of both lower limbs were still present on EMG. A noteworthy decrease in SAA protein to 82 mcg/mL was also observed.

In the hematology evaluation, bone marrow aspiration results exhibited the presence of plasma cells in only 1%, and the flow cytometry analysis showed no significant increase in aberrant plasma cells. These mentioned results indicate the positive response across different systems after two cycles of the given treatment protocol. Table 1 shows the various organ assessment in the pre- and post- 2 cycles of treatment.

**Table 1:** Various organ assessments in pre-treatment and post-treatment phases. NT-proBNP: N-terminal pro-B-type natriuretic peptide; EMG: electromyogram; SAA: serum amyloid A; FLC: free light chain.

	Specifics	Pre-treatment	Post two cycles of treatment	References
Hematology assessment	Serum protein electrophoresis and immunofixation	Presence of monoclonal peak of free lambda type in the beta region 1.4 g/L	Presence of a small monoclonal band of kappa type in the gamma region 1 g/L	-
	FLC lambda	215 mg/L	16.93 mg/L	5.71-26.3 mg/L
	SAA	300 mcg/mL	84 mcg/mL	0-10 mcg/mL
	Bone marrow aspiration and medullogram	Presence of phenotypically suspicious plasma cells (12%)	1% Plasma cells	-
Kidney assessment	Urine protein electrophoresis	Bence Jones proteinuria of free lambda type	Mixed proteinuria is noted with predominance of glomerular proteinuria	-
	Urine protein immunofixation	Not Done	Absence of Bence Jones proteinuria	-
	Urine analysis	Protein +++	Protein +	Negative protein
	24-hour urine collection for proteinuria	750 cc/24 hours with 4,020 mg protein	650 cc/24 hours with 2,303 mg protein	Severely increased: >500 mg/24 hours
	Albumin	1.5 g/dL	2.2 g/dL	3.5-5 g/dL
Cardiac assessment	Echocardiography	Mild concentric hypertrophy with normal-sized cavity and mild pericardial effusion	Left ventricle with normal size Mild pericardial effusion	-
	Troponin	0.338 ng/mL	0.106 ng/mL	0-0.01 ng/mL
	NT-proBNP	7166 pg/mL	5399 pg/mL	<500 pg/mL
	BNP	453 pg/mL	165 pg/mL	<100 pg/mL
Pulmonary assessment	Chest X-ray and CT scan	Bilateral pleural effusion	Less serious bilateral pleural effusion	-
Neurological assessment	EMG	Severe bilateral lower limbs neuropathy	Severe neuropathy more severe in left lower extremity	-

## Discussion

AL amyloidosis is classified as the most common type of amyloidosis, a rare disorder where abnormal light chain proteins accumulate and deposit in organs and tissues, causing multi-organ damage. When diagnosed at an advanced stage, as in the case of this patient, systemic light chain amyloidosis is associated with an unfavorable long-term prognosis. While the average survival rate has improved for AL amyloidosis overall, with the median overall survival increasing from 1.4 years in the 1980s to 4.6 years in the 2010s and a current five-year overall survival rate of approximately 48%, the prognosis remains very poor for those with advanced cardiac involvement, with a median survival of only nine months [9-11]. These patients remain highly susceptible to infections and the occurrence of multiple organ failure [4].

However, the introduction of the ability to detect circulating FLCs has significantly transformed the approach to AL amyloidosis treatment [12]. This advancement permits early disease detection and enhanced prognostic assessment of patient outcomes, thereby offering the potential for improved management and better clinical results. Elevated levels of FLC at the time of diagnosis suggest the clinical implications manifested among the patient. Higher FLC levels exhibit a greater burden of plasma cells, as shown in bone marrow aspiration. Additionally, it could explain gastrointestinal involvement, such as gastroparesis and recurrent episodes of constipation and severe diarrhea. Furthermore, high FLC levels could suggest renal insufficiency and severe cardiac involvement. Moreover, higher FLC levels could explain the perturbation of cardiac biomarkers, including troponin, NT-proBNP, and BNP.

Of particular importance, empirical evidence emphasizes the clinical relevance of FLC levels as a prognostic indicator and highlights its potential impact on patient outcomes, as the results showed that patients with higher FLC levels (>18.2 mg/dL for lambda disease) face a markedly reduced overall survival, in contrast with those having lower FLC levels, who demonstrated a significantly longer overall survival [13]. In the context of this particular patient, it was observed that not only the FLC levels were elevated, but also the SAA levels were high, contributing to diagnostic challenges. Following a comprehensive assessment, the definitive diagnosis of systemic light chain amyloidosis was established, implying that the elevation in SAA level might be attributed to a profound inflammatory response [14].

Staging of systemic light chain amyloidosis is typically based on cardiac biomarkers and renal involvement, according to the Mayo 2012 staging system (Table 2) [15].

**Table 2:** Prognostic staging system for light chain amyloidosis. NT-proBNP: N-terminal pro B-type natriuretic peptide; dFLC: differential free light chain.

Prognostic variables	Value	Score	Stage based on the prognostic variable risk scores	
NT-proBNP (ng/L) or BNP	≥1,800 ng/L ≥400 ng/L	1	Stage I (risk score = 0)	
			Stage II (risk score = 1)	
Troponin T (ng/mL)	≥0.025 mcg/L	1		
			Stage III (risk score = 2)	
dFLC (mg/L)	≥18 mg/dL	1		
			Stage IV (Risk score = 3)	

Following the NCCN Guidelines for AL amyloidosis (Version 1.2024), the treatment protocol was customized based on the patient's stage of the disease. These guidelines offer a comprehensive approach to the management of AL amyloidosis, including chemotherapy regimens and supportive care strategies (Table 3) [16]. Patients ineligible for hematopoietic cell transplant (HCT) include those with cardiac issues such as arrhythmias, decompensated heart failure, or pleural effusion. Additionally, individuals with orthostatic hypotension unresponsive to medical therapy, factor X deficiency, or extensive gastrointestinal involvement are also considered ineligible [16].

**Table 3:** Treatment of systemic light chain amyloidosis according to stage. HCT: hematopoietic cell transplant.

Primary therapy for HCT-eligible and non-eligible patients		
	Staging	Preferred regimens
No significant neuropathy	Stage I–III	-Daratumumab/cyclophosphamide/bortezomib/dexamethasone-autologous HCT (if eligible)
Significant neuropathy	All stage	- Single-agent daratumumab-melphalan/dexamethasone (if ineligible for HCT)

Regarding the patient’s treatment plan, the amalgamation of established therapeutic protocols, namely CyBorD, along with intravenous administration of daratumumab, has proven to be efficacious. It is essential to highlight that the intravenous route was chosen for daratumumab administration because the subcutaneous form, which is generally preferred due to its easier administration and reduced infusion time, was not available in the country due to the economic and sanitary crisis, which have indeed led to significant supply chain disruptions and financial constraints, making it difficult to obtain the subcutaneous medication. The hematologic response attained through the utilization of the daratumumab IV and CyBorD combination exhibited remarkable depth and rapidity, signifying its notable effectiveness in the clinical context. The efficacy outcomes observed in our patients are noteworthy, aligning with findings reported in studies conducted by Suzuki et al. [17] and Palladini et al. (ANDROMEDA trial) [8]. These studies demonstrated that the combination of daratumumab with CyBorD led to substantial improvements in treatment outcomes compared to the use of CyBorD alone in newly diagnosed systemic light chain amyloidosis patients. This consistency in efficacy results between our patient and the findings of the mentioned studies provides further support for the potential benefits of incorporating daratumumab into the treatment regimen for patients newly diagnosed with systemic light chain amyloidosis.

Following the completion of the initial two induction cycles, remarkable improvements were observed in various organs. Notably, kidney function exhibited enhancement, as evidenced by the increased albumin levels in follow-up tests, indicating an improvement in kidney function alongside a significant reduction in proteinuria. Additionally, pleural effusion decreased, and lower limb edema, orthostatic hypotension, and peripheral neuropathy decreased significantly. The results from serum protein electrophoresis and immunofixation demonstrated an absence of plasma cells, suggesting a potential recovery for the patient. A good response was observed in various organs as outlined by the NCCN guidelines [16]. Notably, kidney function improved, as evidenced by the increased albumin levels in the follow-up test, alongside a significant reduction of more than 30% in proteinuria. Also, a decrease of more than 30% in NT-proBNP suggests a good cardiac response. The results from serum and urine protein electrophoresis are negative, with normalization of FLC levels and ratio suggesting a complete hematologic response according to NCCN guidelines [16]. However, peripheral neuropathy did not improve.

Based on these findings and in accordance with the NCCN Guidelines staging, it is justifiable to state that the patient’s condition progressed from stage IV systemic light chain amyloidosis to stage II after only two cycles of treatment. This significant improvement highlights the rapidity and efficacy of the treatment regimen used. As a result, the patient is anticipated to have a more favorable prognosis and an extended overall survival, although the potential risk of relapse remains unpredictable.

## Conclusions

In conclusion, this case report aims to deepen understanding and raise awareness of systemic light chain amyloidosis, a rare but clinically significant disorder. This report contributes to the evolving landscape of amyloidosis management and confirms the results found on ANDROMEDA trial about the efficacy of daratumumab, in association with CyBorD, to reach a rapid complete hematologic response and improve organ response rate. The findings underscore the importance of early detection and tailored treatment strategies aimed at improving patient outcomes and reducing mortality rates. Moving forward, continued research and collaborative efforts are essential to further optimize therapeutic interventions and enhance the overall prognosis for individuals affected by this challenging medical condition.
